# Impact of Current Technology in Laparoscopic Adrenalectomy: 20 Years of Experience in the Treatment of 254 Consecutive Clinical Cases

**DOI:** 10.3390/jcm12134384

**Published:** 2023-06-29

**Authors:** Giovanni Conzo, Renato Patrone, Luigi Flagiello, Antonio Catauro, Alessandra Conzo, Chiara Cacciatore, Federico Maria Mongardini, Giovanni Cozzolino, Rosetta Esposito, Daniela Pasquali, Giuseppe Bellastella, Katherine Esposito, Ludovico Docimo

**Affiliations:** 1 Division of General, Oncological, Mini-Invasive and Obesity Surgery, Department of Traslational Medical Sciences, University of Campania “Luigi Vanvitelli”, 80131 Naples, Italy; luigiflagiello.93@gmail.com (L.F.); antonio.catauro@unicampania.it (A.C.); aleconzo@hotmail.it (A.C.); cacciatore.chiara@virgilio.it (C.C.); f.mongardini@gmail.com (F.M.M.); giovanni91cozzolino@libero.it (G.C.); rosetta.esposito@policliniconapoli.it (R.E.); ludovico.docimo@unicampania.it (L.D.); 2 Dieti Department, University of Naples Federico II, 80100 Naples, Italy; renato.patrone@istitutotumori.na.it; 3 Division of Hepatobiliary Surgical Oncology, Istituto Nazionale Tumori IRCCS Fondazione Pascale-IRCCS di Napoli, 80131 Naples, Italy; 4 Department of Advanced Medical and Surgical Sciences, University of Campania “Luigi Vanvitelli”, 80100 Naples, Italy; daniela.pasquali@unicampania.it; 5 Division of Endocrinology and Metabolic Diseases, Department of Medical, Surgical, Neurological, Metabolic Sciences and Aging, Second University of Naples, 80138 Naples, Italy; giuseppe.bellastella@unicampania.it; 6 Diabetes Unit, Department of Clinical and Experimental Medicine, Second University of Naples, 80138 Naples, Italy; katherine.esposito@unicampania.it

**Keywords:** laparoscopic adrenalectomy, pheochromocytoma, minimally invasive adrenalectomy, Cushing’s syndrome, incidentaloma, energy-based devices, ultrasonic scissors, hemostatic agents

## Abstract

Background: Laparoscopic adrenalectomy (LA), which avoids large abdomen incisions, is considered the gold standard technique for the treatment of benign small- and medium-size adrenal masses (<6 cm) and weighing < 100 g. A trascurable mortality and morbidity rate, short hospitalization and patient rapid recovery are the main advantages compared to traditional surgery. During the past decade, a new surgical technology has been developed that expedites a “clipless” adrenalectomy. Here, the authors analyze a clinical series of 254 consecutive patients who were affected by adrenal gland neoplasms and underwent LA by the transabdominal lateral approach over the two last decades. A literature review is also presented. Methods: Preoperative, intraoperative and postoperative data from 254 patients who underwent LA between January 2003 and December 2022 were retrospectively collected and reviewed. Diagnosis was obtained on the basis of clinical examination, laboratory values and imaging techniques. Doxazosin was preoperatively administered in the case of pheochromocytoma (PCC) while spironolactone and potassium were employed to treat Conn’s disease. The same surgeon (CG) performed all the LA and utilized the same laparoscopic transabdominal lateral approach. Different dissection tools—ultrasonic, bipolar or mixed scissors—and hemostatic agents were used during this period. The following results were obtained: 254 patients were included in the study; functioning tumors were diagnosed in 155 patients, 52 patients were affected by PCCs, 55 by Conn’s disease, 48 by Cushing’s disease. Surgery mean operative time was 137.33 min (range 100–180 min) during the learning curve adrenalectomies and 98.5 min (range 70–180) in subsequent procedures. Mean blood loss was respectively 160.2 mL (range 60–280) and 96.98 mL (range 50–280) in the first 30 procedures and the subsequent ones. Only three conversions (1.18%) to open surgery occurred. No mortality or postoperative major complications were observed, while minor complications occurred in 19 patients (3.54%). In 153 out of 155 functioning neoplasms, LA was effective in the normalization of the endocrine profile. According to our experience, a learning curve consisting of 30 cases was identified. In fact, a lower operative time and a lower complication rate was reported following 30 LA. Conclusions: LA is a safe procedure, even for masses larger than 6 cm and PCCs. Undoubtedly, the development of surgical technology has made it possible reducing operative times, performing a “clipless” adrenalectomy and extending the indications in the treatment of more complex patients. A multidisciplinary team, in referral high-volume centers, is recommended in the management of adrenal pathology. A 30-procedure learning curve is necessary to improve surgical outcomes.

## 1. Introduction

The minimally invasive adrenalectomy (MA), laparoscopic transabdominal or retroperitoneoscopic approach, in absence of local invasion, is the treatment of choice for adrenal masses inferior or equal to 6 cm in diameter, as confirmed by the European Society of Endocrinology Clinical Practice Guideline [1]. Even if in the last decade the adrenalectomy rate remarkably increased in USA, United Kingdom and France [2], it remains a rare operation especially for synchronous bilateral neoplasms (about 5% of adrenal pathologies), performed with the same results by urologists, endocrine and general surgeons. A rate of 14 adrenalectomies for 100,000 discharges (0.014%) was calculated in the USA and 14 adrenalectomies were performed per year in a high-volume center [3]. According to Shariq et al., as part of a wider range of different procedures, same day discharge is possible [4]. Over the years, laparoscopic adrenalectomy (LA) was expanded in the pediatric population, including in a 14-year-old child, with surprising results in removing masses, frequently neuroblastomas, thanks to improvements in experience and endoscopic tools [5].

In the case of larger neoplasms, the laparoscopic approach might be feasible or remain a very useful diagnostic tool while a traditional “open” operation is recommended, particularly in patients with a suspected malignancy. Debate is still open and also characteristics of the mass, body mass index and surgical experience are prognostic factors in the surgical success rate. According to the literature data, LA is therefore the gold standard for the treatment of functioning and non-functioning neoplasms > 3 cm of diameter, weighing 100 g [6,7,8,9], while the removal of masses larger than 6 cm is frequently associated with a higher morbidity rate and longer length of hospital stay [10,11].

Retroperitoneoscopic adrenalectomy, first described by Wantz and Coll, in expert hands, showed similar results of LA, in avoiding the mobilization of abdominal organs. Nevertheless, in case of primary adrenocortical cancer, open surgery is recommended to avoid cell spillage and neoplastic seeding with better oncological margins and outcomes in terms of local relapse and overall survival [12]. On the contrary, LA for adrenal metastatic cancer is nowadays a widespread and widely practiced procedure [6,13,14].

Moreover, during the last two decades, a wide range of innovative surgical instruments, such as ultrasonic, bipolar or integrated radiofrequency scissors, stapler devices and hemostatic agents, have been utilized as a way to reduce operative time and expand the surgical indication with a similar safe rate [15]. Even if major vessel lesions—renal artery or vein, splenic artery and vein and vena cava—have been reported as possible surgical complications, adrenal vessels are small, generally ranging between 3 and 5 mm in diameter, and so intraoperative hemorrhage is not frequent. Nevertheless, the introduction of new energy-based dissection and hemostasis facilitates the dissemination of safe “clipless” adrenalectomy.

Finally, in recent years, robotic adrenalectomy (RA) has also been practiced and the debate is still open regarding its comparison with MA [16].

With the attempt to examine the safety of MA in patients with adrenal neoplasms > 6 cm, the role of new technology and the management of intraoperative complications, the authors retrospectively examined data on 254 patients undergoing LA with the aim to assess technique safety, patient outcomes and the impact of new surgical tools. A literature review was also performed.

## 2. Materials and Methods

The authors reviewed the literature data about the role of LA in the treatment of adrenal neoplasms. So, they analyzed their experience about adrenal pathologies over the last 20 years. All clinical cases of patients undergoing LA for adrenal neoplasms between January 2003 and December 2022 were retrospectively examined.

The inclusion criteria were the following:

- ASA score ≤ III;
- Age less than 80 years;
- Benign functional adrenal tumors up to 8 cm;
- Non-functional tumors ≤ 12 cm;
- Adrenal metastases < 6 cm.

The exclusion criteria were the following:

- Patients with suspected primary malignant disease, based on preoperative imaging examinations;
- Patients treated in open surgery;
- Patients with definitive primary malignancy pathology.

Patient assessment included preoperative, intraoperative and postoperative data. The surgical procedures were all performed by the same surgeon (CG), who was an expert in endocrine and laparoscopic surgery, and assisted by a collaborative team experienced in minimally invasive surgery.

### 2.1. Preoperative Management

All patients underwent prophylactic drug treatment with heparin sodium 4000 U.I. s.c. as antithrombotic prophylaxis. All pheochromocytoma (PCC) patients were given 2 mg of doxazosin up to 3 cp/day until a maximum blood pressure of 160/90 mmhg was reached for at least 24 h and a mean maximum heart rate of 100 BPM for at least 24 h was reached. If these therapeutic objective values were not achieved, 1 compress of 50 mg per day of Atenolol was added in the preoperative work up. Plasma was also expanded with crystalloid solutions. Patients with Conn’s disease and low-potassium levels were given 50 mg of spironolactone up to 6 cp/day and 3 mEq/mL i.v. 1 fl/day of potassium aspartate until normal serum potassium values were reached.

### 2.2. Intraoperative Management

All LAs were performed under general anesthesia with the transperitoneal lateral laparoscopic approach following the procedure described in our previous paper [17]. During the first years of experience, a monopolar hook was utilized. Successively, a Harmonic Scalpel (HS) (Ethicon Endosurgery, Inc., Cincinnati, OH, USA), Ligasure vessel sealing system (LS) (Tyco Valleylab, Boulder, 20052 Monza, Italy) and Thunderbeat (TB) (Olympus Europa Se & Co., Hamburg, Germany) were utilized as advanced hemostasis devices, able to seal and cut vessels with the application of different modalities of energy (radiofrequency, ultrasound or both). Fibrin sealent Tissucol—Tesseel (Baxter, Deerfield, IL, USA), Floseal Hemostatic Matrix (Baxter), Hemopatch^®^ (Baxter) and Tabotamp^®^ were also selectively utilized to achieve a better hemostatic control.

The surgical samples were extracted with Endobag, performing a mini laparotomy in the trocar access site, and a drainage of 20 Fr of the caliber was implemented. During surgery, all recordings of systolic blood pressure values > 180 mmHg were considered hypertensive crises, while systolic pressure values < 90 mmHg were considered hypotensive crises. All patients underwent intraoperative antibiotic prophylaxis with 2 g i.v. of cefazolin.

### 2.3. Postoperative Management

A total of 1000 mL of 0.9% NaCl + 1000 mL of electrolyte solution + 500 mL of 5% i.v. glucose solution was the standard treatment for the first day after surgery. All patients were referred to early mobilization and oral feeding on the first postoperative day (ERAS protocol). Drainage was removed on the first or second postoperative day and early follow-up included clinical and laboratory evaluation at 7, 15 and 30 days after discharge. Subsequently, patients underwent six-monthly check-ups.

### 2.4. Statistics

Data are expressed as mean values, unless otherwise specified. Statistical analysis was performed by SPSS 23rd edition (SPSS©, Chicago, IL, USA), using two-step cluster and *t*-test paired functions. Results are expressed as mean ± standard deviation or percentage and significance was assigned with a *p* value < 0.05.

## 3. Results

From January 2003 until December 2022, a large series of 254 patients, 103 male and 151 female, were enrolled in our study. The average age was 53.2 years (range 22–79). Many of them had cardiovascular and respiratory diseases, as listed in Table 1. In 109 patients, the lesion was located on the left side (42.91%), on the right side in 142 patients (55.9%) and its presence was bilateral in only 3 patients (1.18%). A functional component was diagnosed in 155 patients (61.02%), 55 of whom were suffering from Conn’s disease, 52 from pheochromocytoma and 48 from Cushing’s disease. In 66 cases, the neoformation was found during the execution of diagnostic examinations (incidentaloma), while the presence of a myeloma was found in 2 patients. In addition, in eight patients already treated for kidney and colon cancer, monolateral adrenal metastases were detected (Table 2). The average size of the tumor was 5.82 cm (range from 1.1 to 12.7). Following preoperative anesthesia consultations, 98 patients were classified as ASA I, 104 as ASA II and 52 as ASA III. In seven patients, we carried out therapy in the preoperative period in order to normalize the values of blood pressure, heart rate, serum levels of sodium and potassium. The average operating time was 98.5 min (range 70–180) and 96.98 mL (range 50–280) was the average bleeding (no blood transfusions needed). In three cases, intraoperative complications were reported: one splenic and one liver lesion immediately treated with the help of an electrocoagulation instrument and hemostatic substances (Floseal); in another patient, an inferior vena cava small lesion was observed and sutured without open conversion. Three conversions to open surgery (1.18%) were needed (two due to the presence of tenacious adhesions, one for bleeding). In 48 cases (18.89%), hypertensive crises (95% in patients with PCC, 5% in Cushing’s patients) were observed, 34 intraoperative, during adenoma manipulation and 14 at anesthesia induction, while hypotensive crises were reported in only 11 cases (4.33%). The 30-day postoperative morbidity rate was 3.54% (9/254 patients): 4 abdominal wall hematomas, 2 port site hernia, 2 pneumonia, 1 infection of surgical site (Table 3).

The mean hospital stay was 4.1 day (3–10) with a mean follow-up of 43.52 months (4–150). In 98.5% of patients with a functional adenoma, a normalization of hormonal status was reported (153 out of 155 patients).

## 4. Discussion

Incidental diagnosis of adrenal neoplasms, mostly non-functioning adenomas, is frequently reported in up to 7% of patients submitted to radiological studies for other pathologies [18]. As firstly described in 1992 by M. Gagner, LA by a transperitoneal approach, avoiding large abdominal incisions, represents the most popular surgical treatment for adrenal neoplasms. According to the literature data, it is indicated in cases of PCC up to 8 cm, adenomas and incidentalomas up to 6 cm and metastases < 6 cm [19,20,21,22,23].

Lower morbidity (especially thromboembolism) and mortality rates, decreased postoperative pain, better cosmetic results and lower hospital stays than patients treated with open surgery are the main advantages of LA.

Previous laparotomic surgery, highly vascularized neoplasms or involvement of the adjacent anatomical structures are unfavorable factors that may increase the blood loss and morbidity rate but should not be considered as absolute contraindications. In regard to diameter > 6 cm, most authors demonstrated similar perioperative outcomes than was observed in smaller neoplasms, with a trascurable morbidity rate in absence of local infiltration and performed by an expert adrenal surgeon [24]. Old age, male gender, ASA score, high BMI, previous operations, bilaterality of neoplasms, associated procedures, perioperative heath and respiratory failure were associated to prolonged operative time, higher morbidity, more conversion and increased costs [2,25]. They represent preoperative-predictive surgical risk factors.

During the last two decades, the incidence of severe obesity has doubled beginning as an independent risk factor for worse perioperative outcomes (especially associated with Cushing’s disease). Exposure, visualization and dissection, in obese patients, were more difficult and surgical procedures were almost complex. Perirenal fat thickness has unfavorable impact on dissection and operative time. So, in these patients, several studies demonstrated benefits deriving from robotic access [16].

As for the surgical technique in every case, especially in obese patients, a lateral position and a supernumerary trocar is of paramount importance. They allow a safe management of highly vascularized neoplasms or those bigger than 6 cm and to create a wide working space to control bleeding, thus allowing safe dissection. In some cases, an inclination of 90° is frequently recommended for the left adrenal gland to allow a complete mobilization of the spleen and pancreatic tail. Moreover, in patients with voluminous and fibrous neoplasms of the right adrenal gland with tenacious adherence to the vena cava, an additional port allows the safe mobilization of the liver.

Over the years, the authors partially modified their surgical technique and thanks to the introduction and diffusion of new surgical and hemostatic tools, adrenalectomy was facilitated, and the operative time was reduced. Furthermore, according to our experience in left adrenal gland resection, a right position of 90°, a wide mobilization of the spleen and pancreatic tail until the left diaphragmatic pillar is identified and a wide left colon mobilization occurs are the recommended rules. The far left management of the main adrenal vein with a clipless technique by bipolar sealing devices, following a safe renal vein identification, was becoming preferred.

Likewise, in cases of wide right neoplasms in PCC or Cushing’s patients, a complete hepatic mobilization by cutting the right triangular ligament was performed to expose the vein cava and safely dissect the middle adrenal vein. Moreover, in selected cases, an additional port to insert an additional retractor was suggested.

More recently, retroperitoneoscopy has been the preferred route by urologists and expert surgeons in referral centers, producing the same results, thanks to the absence of intraperitoneal organ mobilization and correlated morbidity [26,27]. However, the approach popularized by Walz, especially in operated patients, to avoid the repositioning of the patient during the procedure, might be preferred in the case of neoplasms < 6 cm of diameter and bilateral neoplasms. According to Japanese Shogo Inoue [28], posterior access was associated to the best cosmetic outcome and patient satisfaction. Nevertheless, literature trials failed to demonstrate superiority of one of the mentioned approaches and thus the choice has been based on surgeon experience [29]. In addition, according to the American Society of Gastrointestinal Endoscopic Surgery, the preferred surgeon route is the best adrenal approach. Nevertheless, transabdominal surgery is preferable in the case of diameter > 6 cm and obese patients, and conversely, retroperitoneal adrenalectomy is beneficial in the case of previous abdominal surgery with a better operative time and lower complication rate.

Additionally, in recent years, we have assisted in robotic diffusion that can improve the quality of vision, but the absence of randomized prospective studies does not allow us to draw definitive conclusions.

Three-dimensional optic systems, robotic arms and movements associated with more precision without a surgeon’s tremor, and more comfortable surgical positions were the main reported and well-known advantages. During the first years of experience with different robotic systems, improved operative time and costs were underlined. In most cases, RA did not add specific advantages even if an operative time similar to that observed in LA was reported after a learning curve [16]. In a metanalysis of 1162 patients, Economopoulos et al. reported similar operative time, complications and conversion rates, concluding that robotics did not ameliorate surgical performance [30,31]. Recently, the EUROCRINE retrospective observational database, based on a large multicentric study of 1005 patients, concluded that RA resulted in shorter hospitalization and a lower complication rate [32].

In 2002, Bennet and Ray introduced hand-assisted LA with the aim to reduce difficulties and complications following the dissection and manipulation of masses > 6 cm, pheochromocytoma or cancer, to avoid capsular effraction and tumor spillage [33]. The technique providing tactile feedback offers several advantages derived from the use of fingers to help and speed up the procedure. The hand-assisted approach did not find a large diffusion probably because the introduction of the hand in the peritoneal cavity may reduce operative space. The absence of randomized trials does not allow us to draw definitive conclusions [34].

Finally, several authors underlined the role of intraoperative indocyanine green fluorescence, especially during conservative partial adrenalectomies [35,36]. The technique can help to recognize glandular anatomy and reduce blood loss. Partial adrenalectomy, performed in case of bilateral procedures in hereditary diseases such as PCCs, to avoid adrenal insufficiency and complications of long-term steroid replacement, was firstly introduced by Irvin in 1983. Due to its ability to maintain omolateral gland function and guarantee a low-recurrence rate, it has gained more interest during recent years [37]. Its promising results were successively confirmed by several papers [38,39,40].

The authors report on the results from their experience and the reviewed literature data focusing on the efficacy of LA by a transperitoneal approach in the treatment of adrenal pathologies. The increasing use of new energy-based dissection and hemostasis devices—ultrasonic scissors, radio-frequency devices and integrated energy devices—speed up operations, often without clip utilization, guaranteeing safety, especially in cases of complex operations or associated procedures [41]. According to our experience, in several patients, “Clipless” or “sutureless” LA simplified procedures reducing operative time. In the same way, novel hemostatic agents (Floseal, Hemopatch, tisseal) have been proved to be very useful in reducing postoperative bleeding “near to zero”. Nevertheless, in our series, before hemostatic agents’ era, no patients underwent reoperation for bleeding.

Regarding surgical skills, to reduce morbidity rate, Pattou maintained that lesions > 4.5 cm in diameter must be treated by experienced surgeons; nevertheless, large indeterminate adrenal neoplasms, carcinoma, a tumor size > 6 cm and the need for concomitant surgical procedures are considered predictive factors for the 30-day morbidity [4,42]. According to the literature data, the most important 30-day morbidity risk factor is considered a tumor size > 6 cm, and open surgery (OS), especially to avoid unfavorable neoplastic seeding, as still recommended [22,43,44,45,46,47,48,49].

On the other hand, adrenocortical carcinoma represents a very rare tumor with an incidence of 1–2 per million per year. The risk of malignancy grows as the size increases, which is about 1% for tumors in diameter < 4 cm, 6% for tumors up to 4–6 cm in diameter and rises to 20% in cases of tumor size > 6 cm; although ACC is rare, it is highly aggressive [31]. Even though family history, virilizing features, mixed hormonal secretion, rapid enhancement and specific MRI contrast pictures are often associated with malignancy, only local invasion or metastases are recognized signs of adrenal cancer. Complete surgical resection is the only possible cure and MA, considering the poor surgical outcomes and the high risk of peritoneal dissemination (cellular spillage, positive surgical margins) during operations, is still a matter of debate. Undoubtedly, a poor prognosis is reported with an overall 5-year survival ranging between 15 to 60% and long-term survival remains dismal because diagnosis is performed in metastatic patients in about 40% of cases [50]. In regard to surgical approach, retrospective studies are associated with conflicting conclusions. The literature data do not support the oncological safety of a minimally invasive approach, confirming open surgery as the gold standard for stages III and IV recommending cancer management in referral centers across the world [51]. Adrenal neoplasms larger than 6 cm may be operated on by a laparoscopic approach but in cases of intraoperative suspects of locoregional infiltration, a prompt conversion is needed. In every case, an accurate preoperative instrumental work-up is of paramount importance, highlighting radiological signs of locoregional infiltration. A trend toward LA in adrenal cancer is nevertheless observed but future prospective randomized trials are necessary for referrals in experienced centers.

According to IPEG (International Paediatric Endosurgery Group) guidelines, a laparoscopic approach is not contraindicated in cases of lesions > 6 cm, and moreover, cancer is not an absolute contraindication if oncological rules are respected [6].

Contrarily, adrenal metastasectomies showed a similar outcome to open surgery if complete resection without capsular effraction and a clear margin of control was achieved [44,52]. Therefore, MA is accepted worldwide as the gold standard in cases of operable adrenal secondaries. In cases of PCC treatment, LA presented the same advantages considered at high risk for the hemodynamic disorders and probably the most insidious adrenal neoplasm. In our experience, a preoperative selective adrenergic blockade with doxazosin did not prevent intraoperative hypertensive episodes, but major related cardiovascular complications—cerebral vascular accident, pulmonary edema, myocardial infarction or ischemia, cardiac arrhythmias and multiorgan failure—were not observed, although in some patients, SBP rose up to 300 mmHg. Even if an intraoperative higher blood loss was mostly observed, a similar surgical morbidity rate was reported in our PCC patients. Several PCCs represent hereditary pathologies such as familial paragangliomas, von Hippel Lindau or MEN 2 syndromes and genetic tests are very useful in preoperative work-up, according to the literature data. In these cases, a multidisciplinary team composed of endocrinologists, geneticists and endocrine surgeons is recommended.

The conversion rate was 1.2% and the presence of adherence in two patients along with hemorrhage were the determining factors. In our series, a mean operative time of 98.5 min (range 70–180) and intraoperative blood loss of 96.98 mL (range 50–280) were similar to that reported by referral centers (54). Surgical experience—laparoscopic and endocrine—is the most important factor, and a learning curve of 30 cases is generally recommended [16,26,27].

According to our experience, a higher operative time was reported in obese patients but without statistically significant differences. As previously reported, 40 cases are considered necessary to mastering minimally invasive procedures, and in our series, undoubtedly new dissecting instruments and hemostatic tools were very useful to speed up safe operations with smaller operative times (15). Additional ports must be adopted to terminate procedures, especially in cases of intraoperative complications, sometimes due to adherences of previous operations and vascular or visceral lesions.

Our retrospective study has some limitations. It is a retrospective study with data from a monocentric database but, in way to minimize the difference, all selected patients were operated on by the same surgeon—expert in endocrine and minimally invasive surgery in a referral center—by a lateral transperitoneal approach and no comparison with an open series was performed. Moreover, all patients were referred to our institution by expert endocrinologists and following a complete and exhaustive preoperative work-up. In our experience, LA was reserved both for tumors larger than 6 cm and for metastases thanks to technological and surgical improvements such us ultrasound, radiofrequency, integrated energy dissection, hemostatic solution or patches (Floseal and Hemopatch Baxter) [17]. In a previous study, we compared different available advanced energy devices in LA highlighting several advantages and concluding that a responsible use of advanced energy devices can improve surgical outcomes guaranteeing a cost savings and patient satisfaction [15].

## 5. Conclusions

LA is also a safe operation in the treatment of large adrenal lesions in which a careful patient selection and adrenal surgery experience may ensure the feasibility and safety of operations with similar medium long-term outcomes than that reported in cases of lesions < 6 cm in diameter [17]. An additional port, especially in cases of perioperative complications, must be recommended. In every case, the correct patient’s position on the operative table is one of the most important concerns. The surgical skill required must be proportional to the tumor size and a multidisciplinary team in the referral center remains of paramount importance to resolve insidious pathologies—Cushing’s disease and PCCs. New surgical technology—dissection and hemostatic tools—helped to reduce operative time, thus simplifying surgical procedures and facilitating the postoperative course. Generally, intraoperative complications may be treated by a minimally invasive approach but sometimes a prompt conversion is preferable to reduce patient risks. Genetic studies are recommended in familial PCCs to obtain a precocious diagnosis. Further randomized and multicentric studies are needed to establish a correct indication of minimally invasive treatment and its role in oncological treatment.

## Figures and Tables

**Table 1:** Demographics (2003–2022).

		Percent
Mean age (years)	53.2 (22–79)	
Male patients (n)	103	40.51
Female patients (n)	151	59.44
Cardiovascular disease (n)	58	22.83
Pulmonary disease (n)	56	22.04
ASA score I–II (n)	202	79.52
ASA score III (n)	52	20.47

**Table 2:** Adrenal neoplasms’ characteristics.

		Percent
Right site (n)	124	55.9
Left site (n)	108	42.91
Bilateral site (n)	3	1.18
Mean size (cm)	5.82 cm	
PCC (n)	52	20.47
Conn’s (n)	55	21.65
Cushing’s (n)	48	18.89
Incidental (n)	66	23.98
Metastasis (n)	8	3.14
Myelolipoma (n)	2	0.78

**Table 3:** Perioperative data.

Mean operative time (min)	98.5 (range 70–180)
Mean Intraoperative blood loss (mL)	96.98 (range 50–280)
Hypertensive crises (SBP > 180 mmHg)	48/254 (18.89%)
Hypotensive crises (SBP < 90 mmHg)	11/254 (4.33%)
Conversion to open procedure (n)	3/254 (1.18%)
Thirty-day morbidity (n)	9/254 (3.54%)
Mean hospital stay (days)	4.1 (range 3–10 days)

## Data Availability

The dataset used and/or analyzed during the current study is available from all individuals from the corresponding author on reasonable request.

## Abbreviations

MA	Minimally invasive adrenalectomy
PCC	Pheochromocytoma
LA	Laparoscopic adrenalectomy
